# Burden of injuries in Vietnam: emerging trends from a decade of economic achievement

**DOI:** 10.1136/injuryprev-2019-043352

**Published:** 2020-01-08

**Authors:** Quynh Anh Nguyen, Thu Ha Nguyen, Justin Beardsley, Chris D Castle, Anh Kim Dang, Zachary V Dingels, Jack T Fox, Chi Linh Hoang, Sonia Lewycka, Zichen Liu, Ali H Mokdad, Nhung Thi Trang Nguyen, Son Hoang Nguyen, Hai Quang Pham, Nicholas L S Roberts, Dillon O Sylte, Bach Xuan Tran, Khanh Bao Tran, Giang Thu Vu, Spencer L James, Thanh Huong Nguyen

**Affiliations:** 1 Department of Health Economics and Finance, Hanoi University of Public Health, Hanoi, Vietnam; 2 Marie Bashir Institute, University of Sydney, Sydney, NSW, Australia; 3 Clinical Research Unit Vietnam, University of Oxford, Ho Chi Minh City, Vietnam; 4 Institute for Health Metrics and Evaluation, University of Washington, Seattle, WA, USA; 5 Institute for Global Health Innovations, Duy Tan University, Hanoi, Vietnam; 6 Center of Excellence in Behavioral Medicine, Nguyen Tat Thanh University, Ho Chi Minh City, Vietnam; 7 Centre for Tropical Medicine and Global Health, Nuffield Department of Medicine, University of Oxford, Oxford, UK; 8 Oxford University Clinical Research Unit, Wellcome Trust Asia Programme, Hanoi, Vietnam; 9 Department of Health Metrics Sciences, School of Medicine, University of Washington, Seattle, WA, USA; 10 Department of Biostatistics, Hanoi University of Public Health, Hanoi, Vietnam; 11 Chronic Disease Epidemiology, Swiss Tropical and Public Health Institute, Basel, Switzerland; 12 Department of Health Economics, Hanoi Medical University, Hanoi, Vietnam; 13 Department of Molecular Medicine and Pathology, University of Auckland, Auckland, New Zealand; 14 Department of Clinical Hematology and Toxicology, Military Medical University, Hanoi, Vietnam; 15 Faculty of Social Science, Behaviors and Health Education, Hanoi University of Public Health, Hanoi, Vietnam

**Keywords:** global, burden of disease, economic development

## Abstract

**Background:**

Vietnam has been one of the fastest-growing world economies in the past decade. The burden of injuries can be affected by economic growth given the increased exposure to causes of injury as well as decreased morbidity and mortality of those that experience injury. It is of interest to evaluate the trends in injury burden that occurred alongside Vietnam’s economic growth in the past decade.

**Methods:**

Results from Global Burden of Disease 2017 were obtained and reviewed. Estimates of incidence, cause-specific mortality, years lived with disability, years of life lost, disability-adjusted life years were analysed and reported for 30 causes of injury in Vietnam from 2007 to 2017.

**Results:**

Between 2007 and 2017, the age-standardised incidence rate of all injuries increased by 14.6% (11.5%–18.2%), while the age-standardised mortality rate decreased by 11.6% (3.0%–20.2%). Interpersonal violence experienced the largest increase in age-standardised incidence (28.3% (17.6%–40.1%)), while exposure to forces of nature had the largest decrease in age-standardised mortality (47.1% (37.9%–54.6%)). The five leading causes of injury in both 2007 and 2017 were road injuries, falls, exposure to mechanical forces, interpersonal violence and other unintentional injuries, all of which increased in incidence from 2007 to 2017. Injury burden varied markedly by age and sex.

**Conclusions:**

The rapid expansions of economic growth in Vietnam as well as improvements in the Sociodemographic Index have occurred alongside dynamic patterns in injury burden. These results should be used to develop and implement prevention and treatment programme.

## Introduction

The burden of injuries in terms of morbidity and mortality can reflect changes in a country’s economy, public health policies that pertain to injuries and healthcare infrastructure. Past research focused on relationships between economy and health outcomes as countries go through epidemiological transitions have been described in detail by Omran and Salomon and Murray.^1 2^ While the premise of the epidemiological transition has been refined in the decades since it was initially proposed, it is evident in contemporary research studies that certain diseases and injuries emerge as important causes of health loss in populations that have experienced socioeconomic growth. Injuries are a unique cause of health loss in that a population might experience both increased and decreased risk of morbidity and mortality from different injuries as an economy improves. For example, an epidemiological study in Thailand in 2007 surmised that young adult mortality in the 1990s was driven by the HIV epidemic but that there was also an increase in road injury deaths attributed to both economic development leading to more exposure to road injuries but also to inadequate prevention measures that may have prevented morbidity or mortality in a higher income country.^3 4^ It is evident that the relationship between economy and injury burden is complex and would benefit from more nuanced analysis in countries with economic expansion.

Vietnam is considered a middle-income economy and in recent years has been noted to demonstrate some of the most rapid economic growth in the eastern Asia region, gaining importance as a contributor to the global economy.^5–9^ This economic expansion has been demonstrated to be associated with declines in fertility and increases in educational attainment,^9^ both expected effects of economic improvement.^10^ Given that Vietnam’s economic growth continues to rise, it is increasingly of interest to measure and understand how its economic and social development have been associated with changes in health outcomes. A country’s burden of injuries is arguably a useful proxy to show the interaction between economic development and health outcomes, since both the incidence and mortality specifically from injuries can be immediately impacted by investment and resource allocation and since accidents and injuries can also be detrimental to economic growth.^4 11–14^ For example, building a highway with resultant high vehicle speeds may increase the risk of a road injury, but building a first response medical system and hospital may decrease the risk of mortality from a road injury.^15^ Additionally, it can be argued that injuries are in general preventable, and therefore, in the setting of economic growth, there is likely value in identifying major contributors to burden in order to plan, invest in, and evaluate infrastructure and health system response measures accordingly. Existing studies on the burden of injury in Vietnam have been focused on injuries from select causes, such as motorcycle accidents, on the economic costs of injury, or are from earlier time periods.^16–18^ It is of interest to provide an updated assessment of injury burden in Vietnam taking into consideration temporal patterns that could have emerged due to economic growth.

The Global Burden of Disease (GBD) study is the most comprehensive effort to date to measure the burden and trends of injury and disease worldwide. It is the product of a global research collaboration that quantifies the impact of hundreds of diseases, injuries and risk factors around the world.^19–21^ The GBD produces annual estimates of all-cause mortality, causes of death, non-fatal health outcomes (ie, incidence, prevalence and years lived with disability (YLDs)), and risk factors. For non-fatal health outcomes, the GBD study uses a method of quantifying health loss in time units adjusted for the relative severity of disability, enabling comparisons over time and across conditions. The GBD study framework measures the burden of all conditions across 195 countries (including Vietnam), for all ages and both sexes, and for years ranging from 1990 to 2017, allowing for a more nuanced analysis of the distribution of disability across demographics and geographies over time and then within different time periods of the study. In this study, we use the GBD 2017 framework and findings to report injury burden in Vietnam from 2007 to 2017.

## Methods

### GBD 2017 study

The GBD 2017 study methods and results have been described in extensive detail elsewhere including description of the analytical estimation framework used to measure deaths, years of life lost (YLLs), YLDs and disability-adjusted life years (DALYs).^19–21^ A detailed overview of the method is provided in online supplementary appendix 1. The methodological components specific to injuries estimation within the GBD framework for Vietnam are summarised as follows. While results for the years from 1990 to 2017 were available, we opted to focus only on the period from 2007 to 2017 for the purposes of this paper.

10.1136/injuryprev-2019-043352.supp1 Supplementary data

### GBD injury classification

Injury incidence and death are defined as International Classification of Diseases, 9th Revision (ICD-9) codes E800-E999 and ICD-10 chapters V–Y, except for deaths and cases of drug overdoses and accidental alcohol poisoning, which are classified under drug and alcohol use disorders. These external cause-of-injury codes or ‘E codes’ codify what caused an injury, for example, a road injury. In terms of the nature-of-injury codes (eg, the lower extremity amputation that can occur from a road injury), injuries were categorised into 47 mutually exclusive and collectively exhaustive nature-of-injury categories using chapters S and T in ICD-10 and codes 800–999 in ICD-9 to quantify the various disabling outcomes of each cause of injury.

### Injury mortality and YLLs

Standard GBD methods for estimating mortality and YLLs were used. These are described in more detail in other GBD literature^22^; a summarised version of this process is described below. First, all available data sources for cause-specific mortality not only from Vietnam but from all locations in the GBD were mapped to the GBD cause list of diseases and injuries. Globally, these data sources consisted of vital registration, verbal autopsy, mortality surveillance, censuses, surveys, hospitals, police records and mortuary data. In Vietnam, only verbal autopsy, survey data and literature studies were available. Second, ill-defined causes of death are redistributed via a process known as garbage code redistribution, which is described in more detail elsewhere.^23 24^ This process ensures that all deaths are accounted for and that every death has one underlying cause assigned. Third, the GBD cause of death ensemble modelling (CODEm) method was used to produce estimates by age, sex, location, year and cause.^25^ CODEm tests a large variety of models to estimate cause of death rates by varying combinations of covariates and modelling techniques and then an ensemble of best-performing models is created based on out-of-sample predictive validity testing. Next, we calculated YLLs by multiplying deaths by the residual life expectancy at the age of death from the GBD 2017 life table.

### Injury incidence, prevalence and YLDs

The detailed approach for estimating non-fatal injury outcomes in GBD is described in GBD literature.^26^ A brief summary of this process is as follows. First, we applied DisMod-MR 2.1 (an epidemiological meta-regression tool that uses a compartmental model framework) to injury incidence data from hospital records and survey data to produce cause-of-injury incidence by location, year, age and sex. For Vietnam, up to the date of analysis, we only had available inpatient admission data for modelling the different injuries, but data sources from other countries and particularly the most proximal countries to Vietnam affect how the models in Vietnam are fit via location random effects. For example, insurance claims records from the Philippines could influence each model fit based on the region random effect pattern as both Vietnam and the Philippines are in the same GBD region. Additionally, we used cause-specific mortality rates from the CODEm process described above and incidence data to compute excess mortality rates in the first month following an injury.

Second, after modelling incidence of each cause-of-injury, we derived a severity hierarchy of nature-of-injury categories to identify the nature-of-injury that would lead to the most severe long-term disability (ie, a combination of likelihood of long-term disability and the corresponding GBD disability weights, which are in summary are measures of the fraction of health one loses with a non-fatal condition) when an individual experiences multiple injuries. To construct the hierarchy, we used data from pooled datasets of follow-up studies in which we translated each individual’s health status measure at 1 year after injury into a disability weight.^27–33^ This process is described in more detail in the GBD literature.^26^

Third, since disability in the event of a cause-of-injury is determined more by the nature-of-injury that results, we estimated the fraction of a given cause that results in a given nature. This process was based on dual-coded (eg, both cause-of-injury and nature-of-injury coded) hospital and emergency department datasets. These proportions were applied to each cause estimate to estimate the distribution of each nature of injury. Additional adjustments were applied to account for duration of injury as well as the proportion of cases that result in permanent disability. The culmination of these steps was the prevalence of each external cause nature of injury combination for each age, sex and year in Vietnam as well as all other locations in the GBD study. To calculate YLDs, the prevalence estimates for each health state resulting from each nature-of-injury were multiplied by a disability weight and corrected for comorbidity with other non-fatal diseases using microsimulation methods. These methods and data sources used are described in more detail elsewhere in GBD literature.^26 34^

### Sociodemographic Index

Sociodemographic Index (SDI) is a unitless index derived from income per capita, average educational attainment over age 15 and total fertility rate under 25. SDI ranges from 0, representing the lowest income per capita, lowest educational attainment and highest fertility observed across 195 GBD locations from 1980 to 2017, to 1, representing the highest income per capita, highest educational attainment and lowest fertility under 25 that are no longer associated with health loss. Additionally, in this paper, we used SDI trends for Vietnam to help quantify socioeconomic development during the time period of this study.

### Uncertainty measurement

Uncertainty is measured at each step of the analytical process based on the sample size, SE or original uncertainty interval from each input to the study. Uncertainty is propagated through each step of the analysis by maintaining distributions of 1000 draws on which each analytical step is conducted. Final 95% uncertainty intervals are determined based on the 25th and 975th value of the ordered values across draws.

### Code and results

Steps of the analytical process were conducted in R, Python and Stata version 13.1. All steps of the analytical process are available online at ghdx.healthdata.org. All results can be downloaded at ghdx.healthdata.org.

### Guidelines for Accurate and Transparent Health Estimates Reporting statement

This study is adherent with guidelines from the Guidelines for Accurate and Transparent Health Estimates Reporting (described in more detail in online supplementary appendix 2).

10.1136/injuryprev-2019-043352.supp2 Supplementary data

## Results

### Sociodemographic development

**Figure 1:**
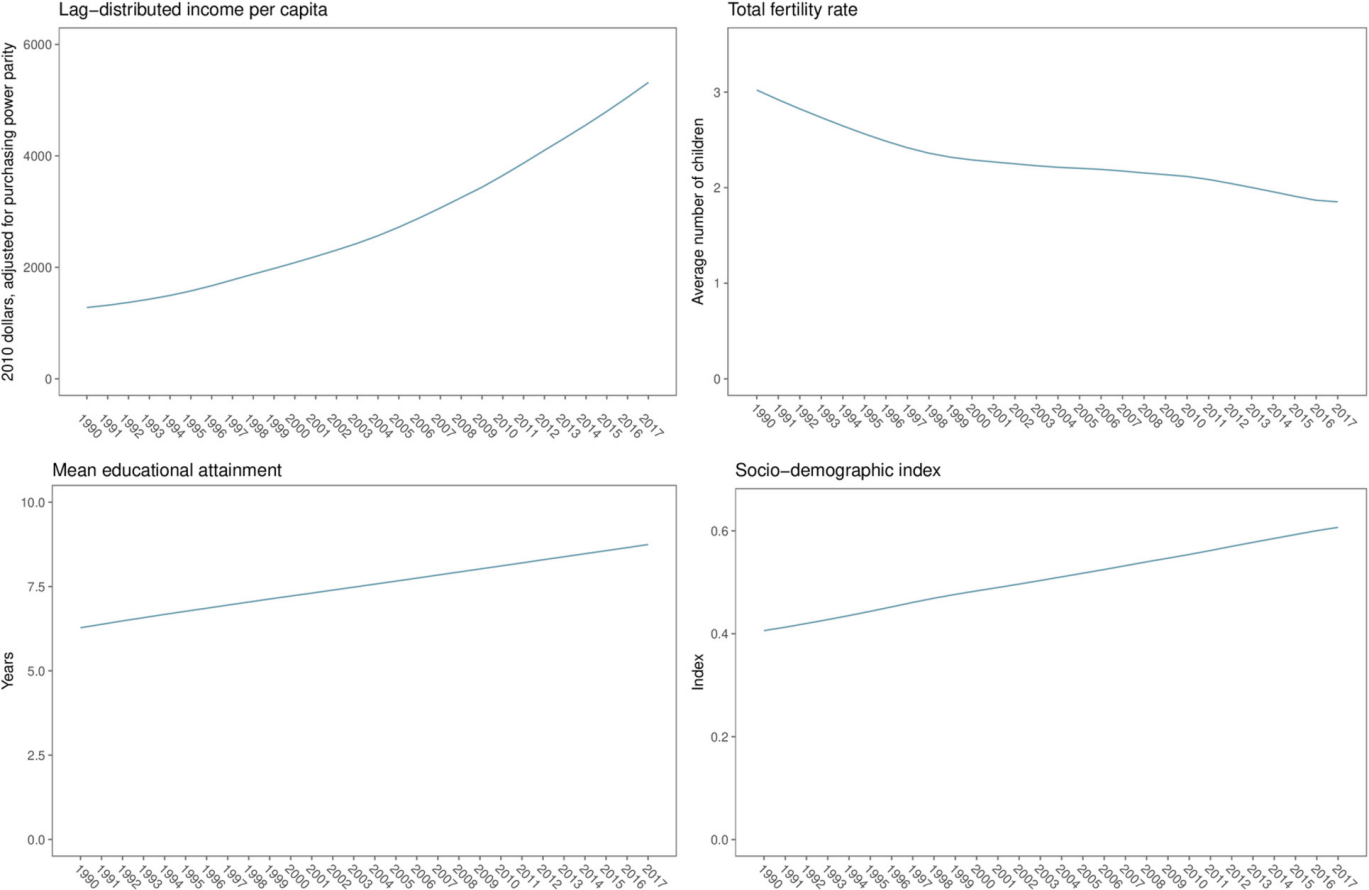
Income per capita, mean educational attainment, total fertility rate, and Sociodemographic Index for Vietnam, 1990–2017.

Figure 1 shows SDI, lag-distributed income per capita (a smoothed Gross Domestic Product (GDP) per capita series), total fertility rate and mean educational attainment for Vietnam between 1990 and 2017. These figures show the estimates derived internally for the GBD study and are based on available data as well as a space-time Gaussian process regression modelling process that incorporate various data sources, covariates and regional patterns in each indicator for GBD 2017. Between 2007 and 2017, the lag-distributed income per capita of Vietnam increased from US$3066 to US$5317 in terms of constant 2010 dollars adjusted for purchasing power parity, representing an 73% increase with a 5.7% annualised rate of increase. During this period, the total fertility rate decreased from 2.17 to 1.85, representing a 14.8% decrease. Average educational attainment increased from 7.8 to 8.7 years, representing an 12% increase. Cumulatively via the SDI derivation process, this resulted in the SDI for Vietnam increasing from 0.53 in 2007 to 0.61 in 2017, representing a 14.0% increase.

### Incidence

**Figure 2:**
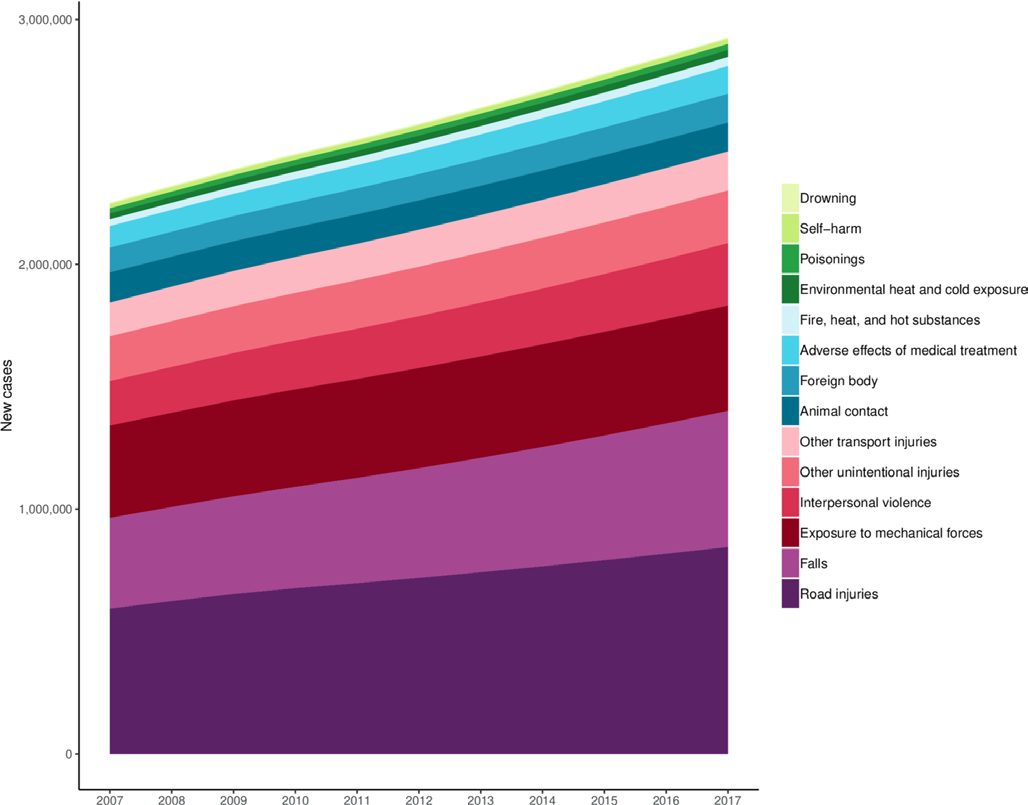
Cause-specific new cases for level 3 injuries, 2007–2017.

Online supplementary appendix table 1 shows the age-standardised incidence rates per 100 000 for each injury in Vietnam as well as the percentage change since 2007. In 2017, the age-standardised incidence of all injuries in Vietnam was 3003 (2851 to 3181) per 100 000 population, which represented an increase of 14.6% (11.5%–18.2%) since 2007. Among all injuries in the third level of the cause hierarchy, the leading cause of injury was road injuries, which had an age-standarised incidence of 821 (718–923) per 100 000 representing a 27.3% (15.7%–39.3%) increase since 2007. The next leading cause of injury was falls that had an age-standardised incidence rate of 592 (520–677) per 100 000, representing a 27.5% (20.9%–34.8%) increase since 2007. Among the road injuries in the more detailed, fourth level of the cause hierarchy, motor vehicle road injuries had the highest age-standardised incidence with 239 (196–287) cases per 100 000, which was a 35.2% (19.8%–50.6%) increase from 2007, which was the greatest of all level 4 causes. Motorcyclist road injuries had the second-greatest incidence in 2017, with an age-standardised incidence of 186 (152–222) per 100 000, which was a 23.6% (8.5%–39.3%) change since 2007. Figure 2 shows new cases for every level 3 injury cause in the GBD cause hierarchy, except for forces of nature, conflict and terrorism, and executions and police conflict, which were observed to have estimates of near zero. This figure shows the sum of new cases of injury across causes as well as the composition of injury burden in terms of absolute number of new cases. The figure also shows how road injuries and falls are two of the important drivers of total new cases of injury across the time period of this study.

10.1136/injuryprev-2019-043352.supp3 Supplementary data

### Cause-specific mortality

Online supplementary appendix table 1 also shows the age-standardised mortality rates per 100 000 for each injury in Vietnam. In 2017, the age-standardised mortality rate for all injuries was 68.2 (60.3–74.6), representing a decrease of 11.6% (3.0%–20.2%) since 2007. Among all injuries in the third level of the cause hierarchy, the leading causes of injury mortality in 2017 were road injuries with an age-standardised mortality rate of 21.4 (18.2–24.2) per 100 000, representing a non-significant decrease of 11.0% (24.1%–1.0%) from 2007 to 2017. The next leading cause was falls, with an age-standardised mortality rate of 18.0 (15.4–21.0) per 100 000, representing an increase of 1.1% (−13.3%–17.2%) from 2007 to 2017. Among road injury subtypes in the fourth level of the cause hierarchy, pedestrian road injuries had the highest cause-specific mortality rate with 8.62 deaths (6.0–11.7) per 100 000 while motor vehicle road injuries had the second highest cause-specific mortality rate with 7.1 deaths (5.0–9.1) per 100 000.

Across all injuries in the level 4 hierarchy, injuries that experienced a significant decline in mortality rates included pedestrian road injuries, poisoning by other means, poisoning by carbon monoxide, other exposure to mechanical forces and unintentional firearm injuries. The remainder of injuries experienced non-significant declines in cause-specific mortality rates. No injury experienced an increase in cause-specific mortality rates.

**Figure 3:**
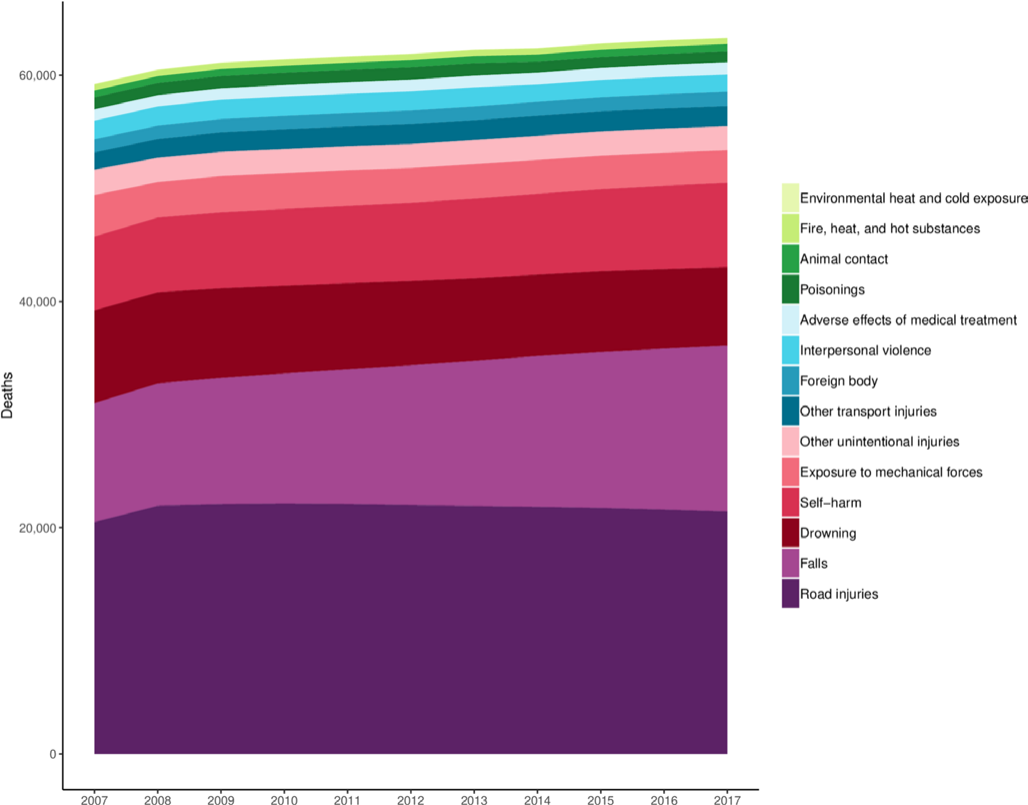
Cause-specific deaths for level 3 injuries, 2007–2017.

Figure 3 shows all-age cause-specific deaths for every level 3 injury cause in the GBD cause hierarchy in Vietnam from 2007 to 2017, except for forces of nature, conflict and terrorism, and executions and police conflict, which were also observed to have estimates of near 0. This figure shows a gradual increase on the number of deaths per year from injuries over the decade, though it is also evident in this figure that the number of deaths per year from road injuries has declined slightly in the more recent years of the study. Similar to figure 2, this figure also demonstrates how road injuries and falls drive a substantial portion of cause-specific mortality of overall injuries in Vietnam.

### YLDs, YLLs and DALYs

**Figure 4:**
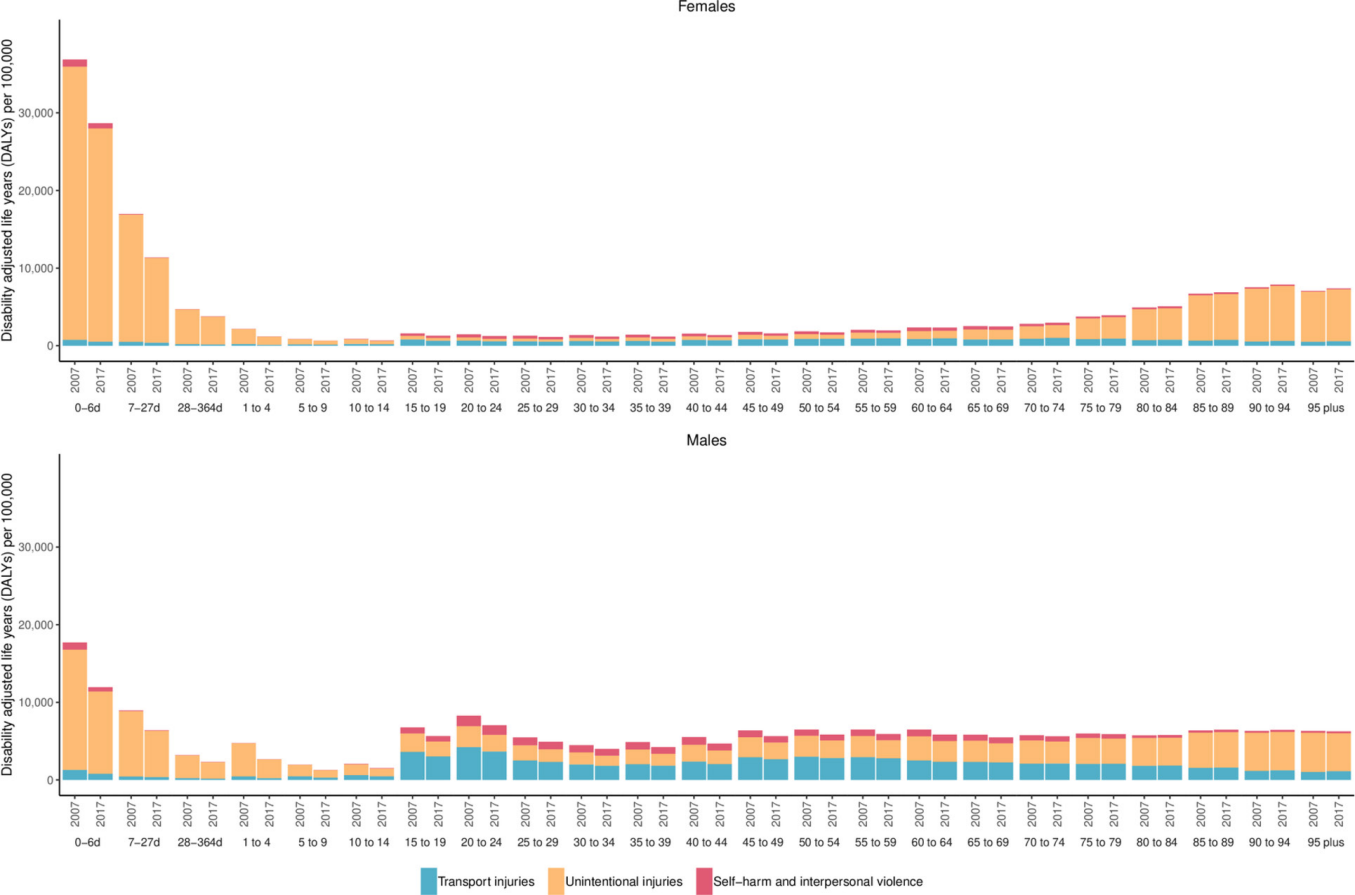
Age-specific and sex-specific DALYs per 100 000 in 2007 and 2017.

Online supplementary appendix table 2 shows the age-standardised YLL, YLD and DALY rates per 100 000 for each injury in Vietnam as well as the percentage change since 2007. Most injuries experienced significant increases in YLDs from 2007 to 2017. Injuries that experienced significant decreases were conflict and terrorism (34.8% (28.9%–40.3%)), drowning (26.1% (23.1%–29.6%)), exposure to forces of nature (24.3% (19.6%–28.9%)), animal contact (12.0% (3.0%–20.3%)), self-harm (8.0% (3.4%–12.7%)), exposure to mechanical forces (4.9% (1.1%–8.6%)) and other unintentional injuries (3.3% (0.2%–6.4%)). Most injuries experienced significant decreases in age-standardised YLL rates between 2007 and 2017. The greatest decreases in age-standardised YLL rates occurred in exposure to forces of nature (47.1% (36.6%–55.8%)), exposure to mechanical forces (36.0% (18.9%–46.5%)), poisonings (29.8% (7.8%–49.7%)) and environmental heat and cold exposure (29.8% (13.8%–43.7%)). In terms of age-standardised DALYs, 10 causes of injuries had significant decreases between 2007 and 2017. The causes where age-standardised DALY rates decreased the greatest were exposure to forces of nature (41.8% (33.6%–49.3%)), conflict and terrorism (34.8% (28.9%–40.3%)), exposure to mechanical forces (32.1% (16.7%–41.8%)), poisonings (28.8% (6.9%–48.5%)), drowning (28.4% (15.8%–38.4%)), other unintentional injuries (20.4% (4.3%–34.6%)), fire, heat and hot substances (20.0% (9.6%–29.55)) and animal contact (16.4% (4.3%–27.0%)). Only falls had a non-significant increase in age-standardised DALY rate, at 0.4% (−11.0%–12.9%). Figure 4 shows age-specific and sex-specific DALYs per 100 000 in 2007 and 2017 for the three level 2 injury causes in the GBD cause hierarchy. This figure demonstrates greater burden in females in early life, driven primarily by unintentional injuries. Males develop a markedly greater burden from injuries in the 1 to 4 age group and experience greater burden though older ages, at which point females again experience greater burden driven primarily by unintentional injuries. The figure also demonstrates that most age and sex groups experienced decreases in DALYs per 100 000 from 2007 to 2017.

10.1136/injuryprev-2019-043352.supp4 Supplementary data

## Discussion

This paper provides an assessment of Vietnam’s injury burden in the setting of its economic development and should be used to inform future injury prevention and treatment interventions. This paper also describes key trends that are germane to improving injury burden in Vietnam. Vietnam has demonstrated remarkable economic and sociodemographic improvement in the past decade.^9^ The improvements in the economy originated from the economic reform in Vietnam in the 1980s and have been mainly attributed to the gradual transitions to a market-based economy, diversifying international relationships, strong foreign investment^35^ and structural change in the economic development towards services and manufacturing.^36^ Meanwhile, declines in fertility and increases in educational attainment have been attributed to economic expansion and to other factors. For example, declines in fertility have resulted from the success of a long-run family planning programme in Vietnam as well as the rapidly urbanisation and the improvement in educational attainment among women.^37^ The increases in educational attainment are also explained by the expansion of education system^38^ as well as the strong parental and child aspirations for education in Vietnam.^39^

This paper explores the patterns in the burden of injuries in Vietnam that has developed alongside these socioeconomic improvements. In summary, we found that while the incidence of many injuries increased significantly, the changes in mortality trended towards declines, though most changes in cause-specific mortality rates were not statistically significant. This relationship, where incidence increased but mortality either did not change or decreased, was true for the overall injuries cause as well as many subtypes of injuries. Every subtype of transport injury experienced a significant increase in incidence but a non-significant decrease in mortality. Poisoning, environmental heat and cold exposure, fires, heat and hot substances, and exposure to mechanical forces all demonstrated significant increases in incidence and significant decreases in mortality, suggesting that despite increased exposures and incidents where these injuries occur, they are not necessarily leading to higher mortality rates. The combination of these trends suggests that case fatality rates among injuries have decreased over the period of this study. This trend may be the result of improved access or quality of healthcare in the event of an injury, which would be consistent with the improvements in Vietnam’s Health care Access and Quality Index, which increased from 51.3 to 60.3 from 2007 to 2017.^40^ In addition, we identified injury burden patterns that merit further research and review. In particular, the markedly higher rate of DALYs from unintentional injuries in the under 1 age groups in females compared with males has not been described in previous injury burden research in Vietnam. More research is needed to understand the basis for these patterns. In addition, we found that road injuries and falls cause a substantial number of cases and deaths per year compared with other injuries, emphasising the importance of further research in preventing these injuries.

There are likely many additional factors that contribute to these trends. The increased incidence for every subtype of transport injury is likely related to the expansion of driving various types of vehicles in Vietnam as driven by population growth and economic expansion. Even if per capita vehicle usage does not change, the increased number of vehicles on the road due to population increases can lead to road hazard. This may be consistent with the large numbers of new cases and deaths we observed from road injuries throughout the period of the study. It is also plausible that these factors have increased the severity of injuries that occur in a given road injury, which could worsen the prospects of survival even in the setting of improved healthcare services. Some improvements in case fatality for different types of injuries may be related to the improvement of tertiary prevention in Vietnam. Three national plans and health sector plans showed the effort of the health sector in improving the community-based first aid and emergency medical services, including the 2002–2010 National Policy for Accident and Injury Prevention (Decision No. 197/2001/QD-TTg of the Prime Minister dated 21 November 2011), the 2011–2015 Injury Prevention Plan in Community of the Health Sector (Decision No. 1900/QD-BYT of Health Minister dated 10 June /2011), and the 2016–2020 Injury Prevention Plan in Community of the Health Sector (Decision No. 216/QD-BYT of Health Minister dated 20 January2017). However, these plans have no clear strategy in terms of primary prevention and secondary intervention, and therefore, the plan for the next period should consider focusing on health education and health promotion to change behaviour, for example, in terms of being compliant with the helmet law, driving while intoxicated, and using mobile phone while driving, as well as creating a safety-oriented environment in terms of accessing quality helmet, using warning signs of dangerous places, improving the safety of the infrastructure via measures such as removing pedestrian crossings at critical intersections.^41–43^ Overall, this requires multidisciplinary efforts rather than only efforts of the health sectors. Future work in injury prevention in Vietnam may benefit from detailed quantitative and qualitative assessments, as has been done for drowning.^44^

As noted in the introduction, injuries are unique in that their mortality rate may be readily amenable to new policies and resources being made available. With road injuries, for example, strictly enforcing a helmet law on motorbike riders or seat belts in car drivers may result in a relatively fast reduction in mortality rates, and adding a first response trauma system to the healthcare infrastructure could improve outcomes within a catchment area.^45 46^ In Vietnam, pertinent policies, which may have impacted injury burden, have included the introduction of a helmet law in December 2007, which may have affected mortality in road injuries,^47^ the Vietnam law on domestic violence prevention and control in 2007,^48^ several drowning prevention programme targeted at children,^49^ and an increasing focus on ensuring suitable living conditions for the elderly, which may impact risk of injurious falls at home,^50^ though there may still be important improvements to address for decreasing risk of falls in this population based on review from the Joint Annual Health Review 2016.^51^ In addition, between 2006 and 2016, there have been significant improvements in the healthcare system in terms of trauma care capabilities, which likely have also contributed to the decreasing mortality-to-incidence ratios (MIRs) by injury that we report in this study.^52^

In the domain of intentional injuries, we reviewed contextual and historical factors, which may have affected the trends we showed in this study. In the case of violence, the incidence of all types of violence-related injury incidence increased while death rates decreased. The introduction and implementation of laws on domestic violence prevention and control from year 2007 may have some effect on lessening the burden of violence in Vietnam. However, the incidence of violence-related injury still increased due to the delayed and ineffective implementation of the law.^53^ In the case of self-harm, there is limited research on this issue. Some research has suggested that misperceptions of mental health problems in Vietnam and lack of mental health services may have been factors in the burden of self-harm injury.^54^

Our study had several limitations. In terms of cause of death modelling, Vietnam lacks vital registration data in the GBD study and the cause of death models instead rely on survey and census data, verbal autopsy studies and modelling based on covariates. It would improve the accuracy and quality in the cause of death modelling process if vital registration data were used instead. The increased uncertainty bounds that arise from using these data sources may also play a role in the lack of a significant change in cause-specific mortality from 2007 to 2017. This may also be a factor in the difference in GBD estimates compared with WHO estimates and official statistics, which reported 24 970 and 8417 deaths, respectively, in 2016 compared with 21 599 deaths in 2016 as reported in GBD 2017.^55^ The difference in these estimates may also be related to the GBD Study’s use of redistribution of ill-defined causes of death and the process by which all cause-specific deaths are scaled to sum to overall mortality, as discussed in the methods section. Conversely, on the side of estimating non-fatal burden, we were able to use the Vietnam hospital data from the Ministry of Health, which provided discharge diagnosis codes for over 71 million admissions across Vietnam in 2012. Using hospital data such as these provide accurate information on diagnosis codes as well as age and sex detail, though we were limited in terms of the number of years available. Additionally, hospital data can be considered biassed if the entire population does not have access to the healthcare system, which may be the case in Vietnam given the large proportion of the population living in rural areas. This limitation in non-fatal data availability may be improved in future GBD cycles on inclusion of the Vietnam National Injuries Survey data. We also examined trends in SDI as part of this paper, and it may be the case that SDI changes experienced by large portions of the population have not been experienced by all areas of the country. Finally, a limitation across the GBD 2017 injuries estimation process is that the longer term sequelae of sexual violence such as post-traumatic stress disorder, depression and anxiety are currently not accounted for in our estimation process.

## Conclusion

The results of this study should be used to inform prevention and treatment programme for injury burden in Vietnam and emphasise the importance of continued data collection and injury burden assessments. Our findings reflect that while more individuals are suffering from injuries in Vietnam, improvements in various prevention approaches, as well as safety and medical care measures, may have resulted in improved outcomes and survival. These patterns are occurring in the setting of a prolonged period of sustained, robust economic growth with improvements in income per capita, educational attainment and total fertility rate. Future improvements in the injury burden in Vietnam will require improved safety measures across multiple domains including road safety, as well as improved first response and medical care systems to decrease mortality rates for injured individuals. It will additionally be important for future policy to continue supporting programme that are contributing to injury prevention and treatment and to also take into account the ageing population in Vietnam that may have increasing susceptibility to certain injuries.

**What is already known on the subject**

- Vietnam has experience rapid economic growth in the recent years.
- There have been injury prevention programme implemented in Vietnam, including a motorcycle helmet law.
- In the Global Burden of Diseases, Injuries and Risk Factors Study 2017, it was reported that there were 63 000 deaths from injuries in Vietnam in 2016 with 2.9 million new cases of injury requiring medical care. To date, these estimates have not been examined and reported in detail with evaluation of the historical and socioeconomic factors that may impact the injury burden in Vietnam.

**What this study adds**

- This is the first study to report morbidity and mortality estimates for every injury in Vietnam from the Global Burden of Disease study cause hierarchy in terms of incidence, cause-specific mortality, years lived with disability, years of life lost and disability-adjusted life years for this period of time.
- This study also incorporates contextual insight in terms of relevant socioeconomic and political developments during the study period that may have impacted injury burden.
