# Supplementary material for: Burden of injuries in Vietnam: emerging trends from a decade of economic achievement
Source: Inj Prev. 2020 Jan 8;26(Suppl 1):i75–82. doi: 10.1136/injuryprev-2019-043352 (PMC7571350; doi:10.1136/injuryprev-2019-043352)
Supplement: Supplementary data [file injuryprev-2019-043352supp001.pdf]

Appendix 1

Summary of General Global Burden of Disease Study Methods

The Institute for Health Metrics and Evaluation with a growing collaboration of scientists produces annual updates of the Global Burden of Disease study. Estimates span the period from 1990 to the most recent completed year (2017). By the time of the release of GBD 2017 in November 2018, there were 3,676 collaborators in 144 countries and 2 territories who contributed to this global public good. Annual updates allow incorporation of new data and method improvements to ensure that the most up-to-date information is available to policy makers in a timely fashion to help make resource allocation decisions.

The guiding principle of GBD is to assess health loss due to mortality and disability comprehensively, where we define disability as any departure from full health. In GBD 2017, estimates were made for 195 countries and territories, and 579 subnational locations, for 28 years starting from 1990, for 23 age groups and both sexes. Deaths were estimated for 282 diseases and injuries, while prevalence and incidence were estimated for 355 diseases and injuries. In order to allow meaningful comparisons between deaths and non-fatal disease outcomes as well as between diseases, the data on deaths and prevalence are summarised in a single indicator, the disability-adjusted life-year (DALY). DALYs are the sum of years of life lost (YLLs) and years lived with disability (YLDs). YLLs are estimated as the multiplication of counts of death and a standard, “ideal”, remaining life expectancy at the age of death. The standard life expectancy is derived from the lowest observed mortality rates in any population in the world greater than 5 million. YLDs are estimated as the product of prevalence of individual consequences of disease (or “sequelae”) times a disability weight that quantifies the relative severity of a sequela as a number between zero (representing “full health”) and 1 (representing death). Disability weights have been estimated in nine population surveys and an open-access internet survey in which respondents are asked to choose the “healthier” between random pairs of health states that are presented with a short description of the main features.

All-cause mortality rates are estimated from vital registration data in countries with complete coverage[1]. For other countries, the probabilities of death before age 5 and between ages 15 and 60 are estimated from censuses and surveys asking mothers to provide a history of children ever born and those still alive, and surveys asking adults about siblings who are alive or have passed away. Using model life tables, these probabilities of death are transformed into age-specific death rates by location, year, and sex.

For cause of death estimation, GBD has collated a large database of cause of death data from vital registrations and verbal autopsy surveys in which relatives are asked a standard set of questions to ascertain the likely cause of death, supplemented with police and mortuary data for injury deaths in countries with no other data[2]. For countries with vital registration data, the completeness is assessed with demographic methods based on comparing recorded deaths with population counts between two successive censuses. The cause of death information is provided in a large number of different classification systems based on versions of the

International Classification of Diseases or bespoke classifications in some countries. All data are mapped into the disease and injury categories of GBD. All classification systems contain codes that are less informative because they lack a specific diagnosis (eg, unspecified cancer) or refer to codes that cannot be underlying cause of death (eg, low back pain or senility) or are intermediate causes (eg, heart failure or sepsis). Such deaths are redistributed to more precise underlying causes of death. After these redistributions and corrections for under-registration, the data are analysed in CODEm (cause of death ensemble model), a highly systematised tool that runs many different models on the same data and chooses an ensemble of models that best reflects all the available input data. Models are chosen with variations in the statistical approach ("mixed effects" of spatiotemporal Gaussian Process Regression), in the unit of analysis (rates or cause fractions), and the choice of predictive covariates. The statistical performance of all models is tested by holding out 30% of the data and checking how well a model covers the data that were held out. To enforce consistency from CODEm, the sum of all cause-specific mortality rates is scaled to that of the all-cause mortality rates in each age, sex, location, and year category.

Non-fatal estimates are based on systematic reviews of published papers and unpublished documents, survey microdata, administrative records of health encounters, registries, and disease surveillance systems[3]. Our Global Health Data Exchange (GHDx, http://ghdx.healthdata.org/) is the largest repository of health data globally. We first set a reference case definition and/or study method that best quantifies each disease or injury or consequence thereof. If there is evidence of a systematic bias in data that used different case definitions or methods compared to reference data we adjust those data points to reflect what its value would have been if measured as the reference. This is a necessary step if one wants to use all data pertaining to a particular quantity of interest rather than choosing a small subset of data of the highest quality only. DisMod-MR 2.1, a Bayesian meta-regression tool, is our main method of analyzing non-fatal data. It is designed as a geographical cascade where a first model is run on all the world's data, which produces an initial global fit and estimates coefficients for predictor variables and the adjustments for alternative study characteristics. The global fit adjusted by the values of random effects for each of seven GBD super-regions, the coefficients on sex and country predictors, are passed down as data to a model for each super-region together with the input data for that geography. The same steps are repeated going from super-region to 21 region fits and then to 195 fits by country and where applicable a further level down to subnational units. Below the global fit, all models are run separately by sex and for six time periods: 1990, 1995, 2000, 2005, 2010, and 2017. During each fit all data on prevalence, incidence, remission, and mortality are forced to be internally consistent. For most diseases, the bulk of data on prevalence or incidence is at the disease level with fewer studies providing data on the proportions of cases of disease in each of the sequelae defined for the disease. The proportions in each sequela are pooled using DisMod-MR 2.1 or meta-analysis, or derived from analyses of patient-level datasets. The multiplication of prevalent cases for each disease sequela and the appropriate disability weight produces YLD estimates that do not yet take into account comorbidity. To correct for comorbidity, these data are used in a simulation to create hypothetical individuals in each age, sex, location, and year combination who experience no, one, or multiple sequelae simultaneously. We assume that disability weights are

multiplicative rather than additive as this avoids assigning a combined disability weight value in any individual to exceed 1, ie, be worse than a "year lost due to death". This comorbidity adjustment leads to an average scaling down of disease-specific YLDs ranging from about 2% in young children up to 17% in oldest ages.

All our estimates of causes of death are categorical: each death is assigned to a single underlying cause. This has the attractive property that all estimates add to 100%. For risks, we use a different, "counterfactual" approach, ie, answering the question: "what would the burden have been if the population had been exposed to a theoretical minimum level of exposure to a risk". Thus, we need to define what level of exposure to a risk factor leads to the lowest amount of disease. We then analyse data on the prevalence of exposure to a risk and derive relative risks for any risk-outcome pair for which we find sufficient evidence of a causal relationship. Prevalence of exposure is estimated in DisMod-MR 2.1, using spatiotemporal Gaussian Process Regression, or from satellite imagery in the case of ambient air pollution. Relative risk data are pooled using meta-analysis of cohort, case-control and/or intervention studies. For each risk and outcome pair, we evaluate the evidence and judge if the evidence falls into the categories of "convincing" or "probable" as defined by the World Cancer Research Fund[4].

From the prevalence and relative risk results, population attributable fractions are estimated relative to the theoretical minimum risk exposure level (TMREL). When we aggregate estimates for clusters of risks, eg, metabolic or behavioural risks, we use a multiplicative function rather than simple addition and take into account how much of each risk is mediated through another risk. For instance, some of the risk of high body mass index is directly onto stroke as an outcome but much of its impact is mediated through high blood pressure, high cholesterol, or high fasting plasma glucose, and we would not want to double count the mediated effects when we estimate aggregates across risk factors[5].

Uncertainty is propagated throughout all these calculations by creating 1,000 values for each prevalence, death, YLL, YLD, or DALY estimate and performing aggregations across causes and locations at the level of each of the 1,000 values for all intermediate steps in the calculation. The lower and upper bounds of the 95% uncertainty interval are the 25th and 975th values of the ordered 1,000 values. For all age-standardised rates, GBD uses a standard population estimated elsewhere in the GBD analytical process.

GBD uses a composite indicator or sociodemographic development, SDI, which reflects the geometric mean of normalised values of a location's income per capita, the average years of schooling in the population 15 and over, and the total fertility rate under age 25. Countries and territories are grouped into five quintiles of high, high-middle, middle, low-middle, and low SDI based on their 2017 values.

1 GBD 2017 Collaborators. Global, regional, and national age- and sex-specific mortality and life expectancy for 195 countries and territories, 1950–2017: a systematic analysis for the Global Burden of Disease Study 2017. *The Lancet* 2018.

2 GBD 2017 Collaborators. Global, regional, and national age-sex-specific mortality for 282 causes of death for 195 countries and territories, 1980–2017: a systematic analysis for the Global Burden of Disease Study 2017. *The Lancet* 2018.

3 GBD 2017 Collaborators. Global, regional, and national incidence, prevalence, and YLDs for 328 acute and chronic diseases and injuries for 195 countries, 1990-2017: a systematic analysis for the Global Burden of Disease Study 2017. *The Lancet* 2018.

4 Food, nutrition, physical activity, and the prevention of cancer: a global perspective. 2007. http://www.aicr.org/assets/docs/pdf/reports/Second_Expert_Report.pdf.

5 GBD 2017 Collaborators. Global, regional, and national comparative risk assessment of 84 behavioural, environmental and occupational, and metabolic risks or clusters of risks for 195 countries and territories, 1990–2017: a systematic analysis for the Global Burden of Disease Study 2017. *The Lancet* 2018.
