# Supplementary material for: Burden of injuries in Vietnam: emerging trends from a decade of economic achievement
Source: Inj Prev. 2020 Jan 8;26(Suppl 1):i75–82. doi: 10.1136/injuryprev-2019-043352 (PMC7571350; doi:10.1136/injuryprev-2019-043352)
Supplement: Supplementary data [file injuryprev-2019-043352supp003.pdf]

Table 1: Age-standardised mortality and incidence rates in 2017 and percentage change from 2007 to 2017 by cause of injury in Vietnam

Cause	Deaths (95% UI)		Incidence (95% UI)	
	2017 age-standardised rates per 100,000	Percentage change in age-standardised rates between 2007 and 2017	2017 age-standardised rates per 100,000	Percentage change in age-standardised rates between 2007 and 2017
**All injuries**	**68.2 (60.3 to 74.6)**	**-11.6% (-20.2% to -3.0%)**	**3 003 (2 851 to 3 181)**	**14.6% (11.5% to 18.2%)**
**Transport injuries**	**23.2 (19.7 to 26.1)**	**-10.3% (-23.0% to 1.4%)**	**1 001 (898 to 1 117)**	**22.9% (13.7% to 32.4%)**
Road injuries	21.4 (18.2 to 24.2)	-11.0% (-24.1% to 1.0%)	821 (718 to 932)	27.3% (15.7% to 39.3%)
Pedestrian road injuries	8.6 (6.0 to 11.7)	-15.9% (-28.8% to -3.8%)	172 (144 to 205)	22.8% (10.8% to 36.1%)
Cyclist road injuries	1.1 (0.7 to 1.8)	6.3% (-13.4% to 29.2%)	145 (115 to 181)	29.2% (16.7% to 44.2%)
Motorcyclist road injuries	4.2 (2.9 to 5.4)	-3.8% (-24.6% to 20.9%)	186 (152 to 222)	23.6% (8.5% to 39.3%)
Motor vehicle road injuries	7.1 (5.0 to 9.1)	-10.8% (-26.3% to 5.7%)	239 (196 to 287)	35.2% (19.8% to 50.6%)
Other road injuries	0.3 (0.2 to 0.6)	-14.9% (-32.7% to 11.7%)	78 (59 to 100)	21.0% (9.3% to 33.5%)
Other transport injuries	1.8 (1.2 to 2.4)	-0.8% (-18.1% to 18.9%)	180 (151 to 215)	6.3% (1.1% to 11.1%)
**Unintentional injuries**	**36.0 (32.2 to 40.1)**	**-13.0% (-21.7% to -4.1%)**	**1 728 (1 629 to 1 827)**	**8.8% (6.1% to 11.9%)**
Falls	18.0 (15.4 to 21.0)	1.1% (-13.3% to 17.2%)	592 (520 to 677)	27.5% (20.9% to 34.8%)
Drowning	7.7 (6.7 to 8.8)	-23.0% (-32.2% to -12.6%)	6 (6 to 7)	-17.0% (-22.8% to -10.3%)
Fire, heat, and hot substances	0.6 (0.5 to 0.7)	-20.5% (-30.7% to -9.7%)	39 (32 to 48)	24.2% (17.4% to 31.7%)
Poisonings	0.9 (0.4 to 1.3)	-29.1% (-49.0% to -7.8%)	24 (19 to 31)	6.7% (-1.8% to 16.2%)
Poisoning by carbon monoxide	0.1 (0.1 to 0.2)	-31.2% (-53.8% to -4.3%)	8 (6 to 11)	6.2% (-5.2% to 17.3%)
Poisoning by other means	0.8 (0.3 to 1.1)	-28.7% (-48.8% to -6.9%)	16 (12 to 21)	7.0% (-2.2% to 16.6%)
Exposure to mechanical forces	2.9 (2.4 to 3.4)	-34.6% (-45.0% to -18.1%)	437 (386 to 494)	3.9% (-1.7% to 10.1%)
Unintentional firearm injuries	0.3 (0.2 to 0.4)	-41.5% (-56.8% to -16.9%)	20 (15 to 26)	-1.3% (-10.5% to 9.1%)
Other exposure to mechanical forces	2.6 (2.2 to 3.1)	-33.6% (-43.4% to -17.8%)	417 (368 to 470)	4.2% (-1.4% to 10.4%)
Adverse effects of medical treatment	1.2 (1.0 to 1.4)	-14.6% (-26.4% to 2.1%)	114 (97 to 133)	4.2% (-1.1% to 10.1%)
Animal contact	0.7 (0.5 to 0.9)	-13.6% (-26.3% to -0.2%)	130 (110 to 154)	-13.3% (-17.1% to -9.3%)
Venomous animal contact	0.5 (0.3 to 0.7)	-13.5% (-27.0% to 3.3%)	56 (45 to 69)	-11.3% (-15.1% to -7.2%)
Non-venomous animal contact	0.2 (0.1 to 0.3)	-13.7% (-30.8% to 5.8%)	73 (60 to 91)	-14.8% (-18.8% to -10.5%)
Foreign body	1.6 (1.4 to 1.9)	-7.8% (-21.7% to 7.5%)	122 (102 to 143)	4.1% (0.9% to 7.8%)
Pulmonary aspiration and foreign body in airway	1.4 (1.2 to 1.7)	-7.4% (-21.8% to 8.8%)	9 (7 to 11)	18.1% (5.5% to 30.4%)
Foreign body in eyes	--	--	77 (58 to 97)	2.3% (-1.7% to 7.3%)
Foreign body in other body part	0.2 (0.1 to 0.3)	-10.7% (-27.6% to 10.7%)	37 (30 to 45)	5.2% (0.4% to 9.4%)
Environmental heat and cold exposure	0.1 (0.0 to 0.1)	-29.0% (-41.2% to -12.6%)	32 (26 to 40)	9.7% (5.5% to 14.0%)
Exposure to forces of nature	0.2 (0.2 to 0.2)	-47.1% (-54.6% to -37.9%)	11 (10 to 13)	-47.1% (-47.1% to -47.1%)
Other unintentional injuries	2.1 (1.6 to 2.4)	-21.6% (-36.8% to -4.1%)	220 (191 to 252)	2.9% (-2.6% to 8.2%)
**Self-harm and interpersonal violence**	**8.9 (7.3 to 10.3)**	**-8.6% (-21.8% to 5.4%)**	**274 (237 to 316)**	**25.7% (16.2% to 36.7%)**
Self-harm	7.4 (6.1 to 8.6)	-6.5% (-18.9% to 6.9%)	21 (18 to 24)	5.0% (-2.1% to 13.8%)
Self-harm by firearm	0.2 (0.1 to 0.3)	-12.9% (-31.3% to 8.7%)	0 (0 to 1)	4.3% (-13.9% to 24.4%)
Self-harm by other specified means	7.2 (6.0 to 8.4)	-6.4% (-18.7% to 7.0%)	20 (18 to 23)	5.0% (-2.2% to 13.8%)
Interpersonal violence	1.5 (1.0 to 2.0)	-17.7% (-40.9% to 7.6%)	251 (213 to 293)	28.3% (17.6% to 40.1%)
Assault by firearm	0.1 (0.1 to 0.2)	-17.2% (-47.9% to 22.3%)	5 (3 to 7)	56.9% (39.4% to 77.7%)
Assault by sharp object	1.0 (0.5 to 1.4)	-16.0% (-42.6% to 15.4%)	70 (54 to 88)	18.0% (4.9% to 32.6%)
Assault by other means	0.4 (0.3 to 0.6)	-21.6% (-46.6% to 5.8%)	176 (149 to 208)	32.2% (21.5% to 44.1%)
Conflict and terrorism	0.0 (0.0 to 0.0)	--	0 (0 to 0)	--

Cause	Deaths (95% UI)		Incidence (95% UI)	
	2017 age-standardised rates per 100,000	Percentage change in age-standardised rates between 2007 and 2017	2017 age-standardised rates per 100,000	Percentage change in age-standardised rates between 2007 and 2017
Executions and police conflict	0.0<br>(0.0 to 0.0)	-9.2%<br>(-38.5% to 35.5%)	3<br>(2 to 4)	-7.1%<br>(-36.7% to 38.1%)
