# Supplementary material for: Burden of injuries in Vietnam: emerging trends from a decade of economic achievement
Source: Inj Prev. 2020 Jan 8;26(Suppl 1):i75–82. doi: 10.1136/injuryprev-2019-043352 (PMC7571350; doi:10.1136/injuryprev-2019-043352)
Supplement: Supplementary data [file injuryprev-2019-043352supp004.pdf]

Table 2: Age-standardised YLL, YLD, and DALY rates in 2017 and percentage change from 2007 to 2017 by cause of injury in Vietnam

Cause	YLLs (95% UI)		YLDs (95% UI)		DALYs (95% UI)	
	2017 age-standardised rates per 100,000	Percentage change in age-standardised rates between 2007 and 2017	2017 age-standardised rates per 100,000	Percentage change in age-standardised rates between 2007 and 2017	2017 age-standardised rates per 100,000	Percentage change in age-standardised rates between 2007 and 2017
**All injuries**	**2 559 (2 220 to 2 820)**	**-17.4% (-27.0% to -7.5%)**	**374 (275 to 494)**	**8.3% (5.1% to 11.5%)**	**2 933 (2 578 to 3 225)**	**-14.8% (-23.8% to -6.0%)**
**Transport injuries**	**1 007 (832 to 1 141)**	**-13.3% (-27.0% to -0.5%)**	**190 (136 to 254)**	**11.9% (8.6% to 15.4%)**	**1 197 (1 022 to 1 341)**	**-10.0% (-22.3% to 1.2%)**
Road injuries	931 (769 to 1 059)	-13.9% (-27.7% to -1.3%)	139 (99 to 186)	18.8% (15.6% to 22.1%)	1 069 (912 to 1 205)	-10.8% (-23.7% to 0.9%)
Pedestrian road injuries	318 (214 to 446)	-20.9% (-34.0% to -8.0%)	36 (25 to 49)	13.7% (10.1% to 17.3%)	354 (248 to 485)	-18.3% (-31.0% to -6.4%)
Cyclist road injuries	46 (27 to 73)	1.7% (-17.3% to 23.4%)	19 (13 to 26)	20.7% (17.0% to 24.9%)	65 (44 to 94)	6.6% (-9.0% to 22.6%)
Motorcyclist road injuries	225 (156 to 287)	-5.7% (-27.4% to 21.0%)	41 (28 to 57)	14.8% (10.7% to 18.9%)	266 (196 to 329)	-3.0% (-22.3% to 19.9%)
Motor vehicle road injuries	328 (234 to 411)	-13.5% (-29.5% to 4.0%)	33 (24 to 45)	30.5% (26.4% to 34.7%)	361 (264 to 448)	-10.7% (-26.2% to 6.0%)
Other road injuries	14 (8 to 24)	-17.5% (-36.1% to 10.3%)	9 (6 to 12)	15.2% (11.3% to 18.7%)	23 (16 to 33)	-7.0% (-24.0% to 11.9%)
Other transport injuries	76 (50 to 108)	-4.0% (-23.0% to 18.8%)	51 (36 to 69)	-3.1% (-6.7% to 0.8%)	127 (98 to 161)	-3.6% (-15.7% to 10.0%)
**Unintentional injuries**	**1 178 (1 040 to 1 318)**	**-22.4% (-31.5% to -13.3%)**	**141 (101 to 190)**	**3.1% (-0.6% to 6.4%)**	**1 320 (1 168 to 1 473)**	**-20.3% (-28.6% to -12.1%)**
Falls	327 (282 to 381)	-2.7% (-15.4% to 11.8%)	59 (42 to 81)	21.1% (17.0% to 24.8%)	387 (338 to 445)	0.4% (-11.0% to 12.9%)
Drowning	433 (367 to 508)	-28.4% (-38.5% to -15.8%)	2 (2 to 3)	-26.1% (-29.6% to -23.1%)	435 (369 to 510)	-28.4% (-38.4% to -15.8%)
Fire, heat, and hot substances	22 (18 to 28)	-25.1% (-37.3% to -11.9%)	13 (9 to 18)	-8.9% (-18.1% to 0.5%)	35 (29 to 42)	-20.0% (-29.5% to -9.6%)
Poisonings	38 (18 to 53)	-29.8% (-49.7% to -7.8%)	2 (1 to 2)	11.1% (-1.6% to 24.4%)	39 (20 to 54)	-28.8% (-48.5% to -6.9%)
Poisoning by carbon monoxide	6 (3 to 9)	-30.7% (-54.3% to -2.9%)	0 (0 to 1)	10.6% (-2.0% to 23.7%)	6 (3 to 9)	-29.0% (-51.9% to -2.3%)
Poisoning by other means	31 (15 to 44)	-29.7% (-50.1% to -7.3%)	1 (1 to 2)	11.3% (-1.4% to 25.5%)	33 (16 to 45)	-28.7% (-48.8% to -7.0%)
Exposure to mechanical forces	130 (108 to 151)	-36.0% (-46.5% to -18.8%)	28 (19 to 38)	-4.9% (-8.6% to -1.1%)	157 (133 to 180)	-32.1% (-41.8% to -16.7%)
Unintentional firearm injuries	15 (10 to 20)	-42.0% (-56.4% to -17.6%)	4 (3 to 5)	-8.3% (-11.9% to -4.9%)	19 (14 to 24)	-37.7% (-50.9% to -16.5%)
Other exposure to mechanical forces	115 (95 to 134)	-35.1% (-45.6% to -18.6%)	24 (17 to 34)	-4.3% (-8.3% to -0.4%)	139 (117 to 161)	-31.3% (-40.9% to -16.6%)
Adverse effects of medical treatment	40 (33 to 48)	-14.4% (-25.6% to -1.9%)	1 (1 to 2)	4.2% (-1.0% to 10.0%)	41 (34 to 49)	-13.9% (-25.0% to -1.8%)
Animal contact	24 (18 to 30)	-16.9% (-29.3% to -3.6%)	3 (2 to 5)	-12.0% (-20.4% to -3.0%)	28 (21 to 33)	-16.4% (-27.0% to -4.3%)
Venomous animal contact	17 (11 to 21)	-17.8% (-29.1% to -3.4%)	2 (1 to 3)	-7.4% (-19.6% to 6.3%)	19 (13 to 23)	-16.7% (-27.2% to -3.8%)
Non-venomous animal contact	8 (6 to 12)	-15.0% (-32.7% to 7.5%)	1 (1 to 2)	-20.7% (-25.0% to -16.8%)	9 (6 to 13)	-15.7% (-31.5% to 3.0%)
Foreign body	59 (46 to 76)	-19.2% (-38.1% to 5.7%)	6 (4 to 8)	-3.5% (-7.7% to 0.7%)	65 (52 to 82)	-18.0% (-35.7% to 5.0%)
Pulmonary aspiration and foreign body in airway	54 (41 to 70)	-19.2% (-38.6% to 7.0%)	1 (1 to 2)	1.1% (-5.3% to 7.9%)	55 (42 to 71)	-18.8% (-38.0% to 6.9%)
Foreign body in eyes	--	--	1 (0 to 1)	1.9% (-1.9% to 6.4%)	1 (0 to 1)	1.9% (-1.9% to 6.4%)
Foreign body in other body part	5 (3 to 8)	-19.9% (-41.0% to 7.0%)	4 (3 to 5)	-6.0% (-10.9% to -1.2%)	9 (7 to 13)	-14.2% (-27.7% to 1.9%)
Environmental heat and cold exposure	2 (1 to 2)	-29.8% (-43.7% to -13.8%)	5 (4 to 7)	-3.4% (-7.5% to 0.8%)	7 (5 to 9)	-12.2% (-19.3% to -5.9%)
Exposure to forces of nature	10 (9 to 12)	-47.1% (-55.8% to -36.6%)	4 (3 to 6)	-24.3% (-28.9% to -19.6%)	15 (13 to 17)	-41.8% (-49.3% to -33.6%)
Other unintentional injuries	93 (71 to 110)	-23.0% (-39.1% to -4.5%)	18 (12 to 25)	-3.3% (-6.4% to -0.2%)	110 (89 to 129)	-20.4% (-34.6% to -4.3%)
**Self-harm and interpersonal violence**	**374 (301 to 435)**	**-10.5% (-24.9% to 4.4%)**	**43 (33 to 54)**	**11.1% (7.8% to 14.9%)**	**417 (343 to 478)**	**-8.6% (-22.1% to 5.1%)**
Self-harm	298 (242 to 345)	-8.1% (-22.3% to 6.5%)	3 (2 to 3)	-8.0% (-12.7% to -3.4%)	301 (244 to 348)	-8.1% (-22.2% to 6.4%)
Self-harm by firearm	8 (4 to 13)	-13.3% (-33.8% to 10.5%)	0 (0 to 0)	-2.1% (-6.5% to 2.6%)	8 (4 to 13)	-13.2% (-33.6% to 10.4%)
Self-harm by other specified means	290 (236 to 336)	-8.0% (-22.3% to 7.0%)	3 (2 to 3)	-8.1% (-12.9% to -3.5%)	293 (239 to 339)	-8.0% (-22.1% to 6.9%)
Interpersonal violence	75 (48 to 103)	-18.8% (-42.9% to 8.7%)	40 (31 to 51)	13.0% (9.5% to 16.8%)	115 (85 to 143)	-10.0% (-27.6% to 10.0%)
Assault by firearm	5 (3 to 9)	-18.1% (-50.1% to 23.5%)	1 (1 to 1)	45.3% (36.9% to 53.9%)	6 (4 to 10)	-11.3% (-41.0% to 25.6%)
Assault by sharp object	51 (28 to 74)	-16.8% (-44.6% to 17.0%)	8 (6 to 11)	10.1% (5.2% to 14.7%)	59 (36 to 82)	-14.0% (-38.9% to 16.3%)
Sexual violence	--	--	15 (10 to 22)	5.8% (1.2% to 10.3%)	15 (10 to 22)	5.8% (1.2% to 10.3%)
Assault by other means	18 (12 to 28)	-24.1% (-49.7% to 3.8%)	16 (11 to 22)	20.3% (14.9% to 25.5%)	35 (26 to 45)	-8.3% (-26.3% to 10.3%)
Conflict and terrorism	0 (0 to 0)	--	0 (0 to 0)	-34.8% (-40.3% to -28.9%)	0 (0 to 0)	-34.8% (-40.3% to -28.9%)
Executions and police conflict	1 (1 to 2)	-8.4% (-38.7% to 39.2%)	0 (0 to 0)	-5.6% (-16.4% to 11.5%)	1 (1 to 2)	-7.8% (-33.0% to 30.3%)
